# Supplementary material for: Using the Prediction Model Risk of Bias Assessment Tool (PROBAST) to Evaluate Melanoma Prediction Studies
Source: Cancers (Basel). 2022 Jun 20;14(12):3033. doi: 10.3390/cancers14123033 (PMC9221327; doi:10.3390/cancers14123033)
Supplement: Supplementary file 1 [file cancers-14-03033-s001.zip › cancers-1731847-supplementary.pdf]

Table S1: Decision rules for high and unclear PROBAST ratings

Domain 1: Participants	
**ROB category**	**Rules**
High	• Case-control studies: hospital controls or cases from only one center<br>• Cohorts: self-selected screening population, no population sampling<br>• Pooled studies and meta-analyses:<br>- If at least one study is included that should be rated as high
Unclear	• No or limited information on the study participants<br>• Pooled studies and meta-analyses:<br>- If no references to the studies included were provided
**Domain 2: Predictors**	
**ROB category**	**Rules**
High	• Pooled studies: default is **high ROB**, as heterogeneity between studies is assumed. Exceptions:<br>- Justification was given that there was no heterogeneity e.g. because identical protocols were used to assess risk factors → **low ROB**<br>- Example from our studies: different assessment of number of nevi (nevi count on arms versus nevi count on whole body), but use of quantiles for risk model instead of absolute nevi counts [1] → **low ROB**
Unclear	• Case-control studies: Use of risk factors for which recall bias is possible (especially risk factors related to UV exposure like “sunburns” and “sunbed use”)<br>• No or limited information on the selection and assessment of predictors
**Domain 3: Outcome**	
**ROB category**	**Rules**

High	• Multiple outcomes, not only melanoma (e.g., "severely dysplastic naevus/cannot exclude melanoma" [2])<br>• Melanoma diagnosis not verified/histological confirmed (e.g. "suspected melanoma" [3])<br>• Self-reported outcome e.g. lifetime melanoma via surveys
Unclear	• No or limited information on outcome
**Domain 4: Analysis**	
**ROB category**	**Rules**
High	• No validation (internal or external)<br>Exception: prespecified models<br>• No performance evaluation<br>• Limited sample size concerning number of predictors
Unclear	• No or limited information on analysis<br>• Components of the analysis whose impact on the results is unclear. E.g., ordinal incorporation of PRS [4], rounding of model coefficients to define the risk score [5], handling of ordinal variable as continuous variable [6]
**Overall ROB (according to the given rules in the PROBAST tool [7])**	
**Low ROB:**	If all domains were rated low ROB.<br>If a prediction model was developed without any external validation, and it was rated as low risk of bias for all domains, consider downgrading to high ROB.<br>Such a model can only be considered as low ROB, if the development was based on a very large data set and included some form of internal validation
**High ROB:**	If at least one domain was judged to be at high ROB.
**Unclear ROB:**	If an unclear risk of bias was noted in at least one domain and all other domains were rated low ROB.

Generally, within the domains, if criteria suggest both a "high" and an "unclear" ROB, the category "high" should be chosen for being more specific.

References

1. Davies, J.R.; Chang, Y.M.; Bishop, D.T.; Armstrong, B.K.; Bataille, V.; Bergman, W.; Berwick, M.; Bracci, P.M.; Elwood, J.M.; Ernstoff, M.S.; et al. Development and validation of a melanoma risk score based on pooled data from 16 case-control studies. *Cancer Epidemiol Biomarkers Prev* **2015**, *24*, 817-824, doi:10.1158/1055-9965.EPI-14-1062.
2. Guther, S.; Ramrath, K.; Dyall-Smith, D.; Landthaler, M.; Stolz, W. Development of a targeted risk-group model for skin cancer screening based on more than 100,000 total skin examinations. *J Eur Acad Dermatol Venereol* **2012**, *26*, 86-94, doi:10.1111/j.1468-3083.2011.04014.x.
3. Goldberg, M.S.; Doucette, J.T.; Lim, H.W.; Spencer, J.; Carucci, J.A.; Rigel, D.S. Risk factors for presumptive melanoma in skin cancer screening: American Academy of Dermatology National Melanoma/Skin Cancer Screening Program experience 2001-2005. *J Am Acad Dermatol* **2007**, *57*, 60-66, doi:10.1016/j.jaad.2007.02.010.
4. Gu, F.; Chen, T.H.; Pfeiffer, R.M.; Fargnoli, M.C.; Calista, D.; Ghiorzo, P.; Peris, K.; Puig, S.; Menin, C.; De Nicolo, A.; et al. Combining common genetic variants and non-genetic risk factors to predict risk of cutaneous melanoma. *Hum Mol Genet* **2018**, *27*, 4145-4156, doi:10.1093/hmg/ddy282.
5. Williams, L.H.; Shors, A.R.; Barlow, W.E.; Solomon, C.; White, E. Identifying Persons at Highest Risk of Melanoma Using Self-Assessed Risk Factors. *J Clin Exp Dermatol Res* **2011**, *2*, doi:10.4172/2155-9554.1000129.
6. Penn, L.A.; Qian, M.; Zhang, E.; Ng, E.; Shao, Y.; Berwick, M.; Lazovich, D.; Polsky, D. Development of a melanoma risk prediction model incorporating MC1R genotype and indoor tanning exposure: impact of mole phenotype on model performance. *PLoS One* **2014**, *9*, e101507, doi:10.1371/journal.pone.0101507.
7. Wolff, R.F.; Moons, K.G.M.; Riley, R.D.; Whiting, P.F.; Westwood, M.; Collins, G.S.; Reitsma, J.B.; Kleijnen, J.; Mallett, S.; Groupdagger, P. PROBAST: A Tool to Assess the Risk of Bias and Applicability of Prediction Model Studies. Ann Intern Med 2019, 170, 51-58, doi:10.7326/M18-1376.

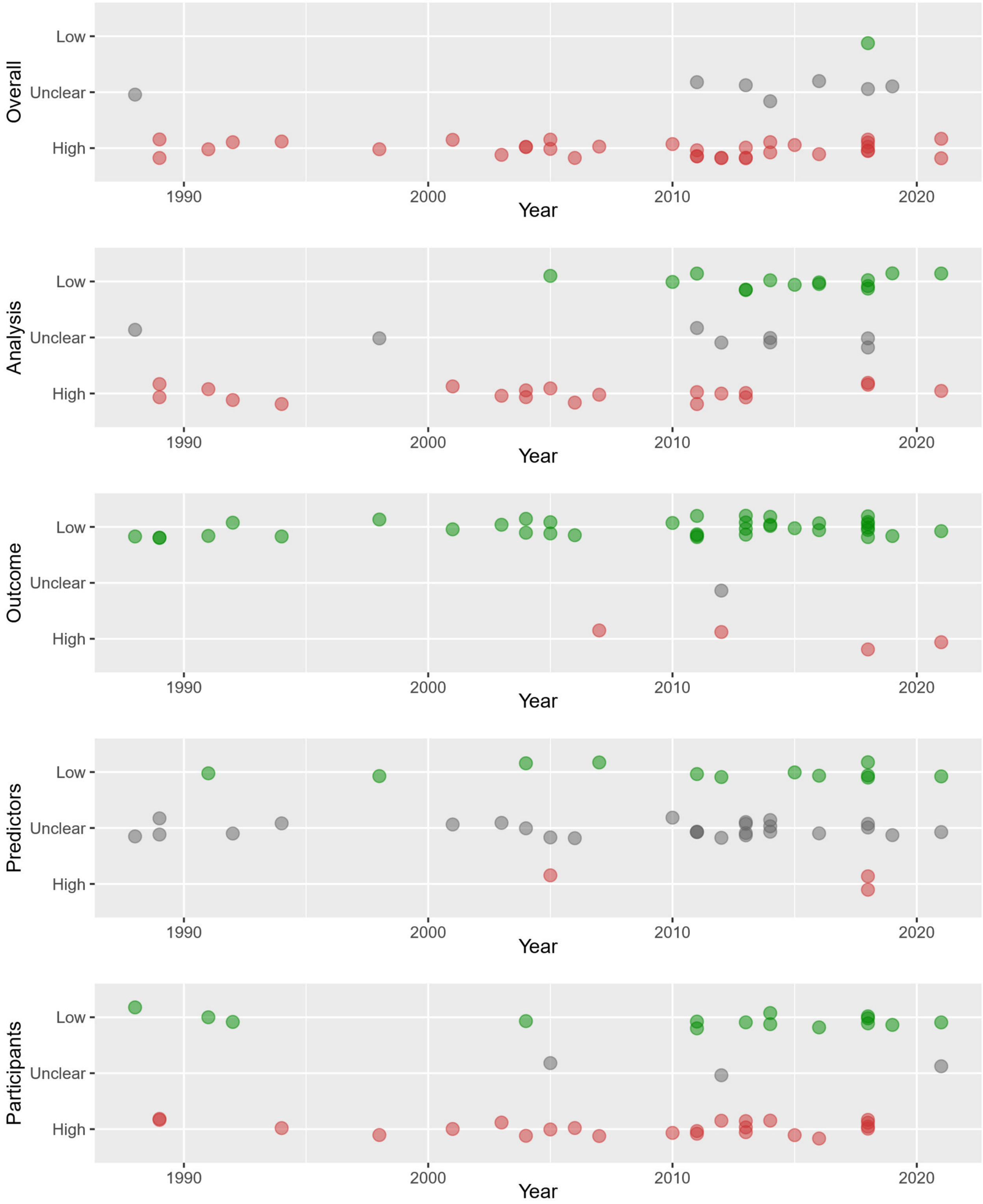

Figure S1: Distribution of overall and domain-specific ROB ratings over time (N=42 studies).
